# Renal denervation revisited; should we pay attention?

**DOI:** 10.1007/s12471-022-01663-1

**Published:** 2022-02-15

**Authors:** R. J. de Winter

**Affiliations:** Department of Cardiology, Heart Center, Amsterdam University Medical Centres, Amsterdam, The Netherlands

In this issue of the journal, L. Feyz and colleagues present the results of a small, randomised study investigating the safety and efficacy of renal denervation (RDN) in patients with heart failure and reduced ejection fraction [1]. Primary safety endpoint was the combination of cardiovascular death, rehospitalisation for heart failure, and acute kidney injury at 6 months. Efficacy was defined as the change in iodine-123 labelled meta-iodobenzylguanidine (^123^I‑MIBG) heart-to-mediastinum ratio at 6 months. The authors demonstrate that RDN with the Vessix V2 Renal Denervation System (Boston Scientific, Natick, MA, USA) was safe, but the primary efficacy endpoint was not different when comparing the RDN treated patients with the patients treated with optimal medical therapy.

The study was small, only 50 patients, less than the anticipated 71 patients. However, collecting these data has been the result of a major endeavour. The study group screened 343 eligible patients over a period of 4 years. Patients randomised to RDN underwent the invasive procedure and all patients underwent MIBG scanning, with subsequent meticulous follow-up for 6 months to assess outcomes. It is important that neutral or negative studies, albeit small, are published for readers to appreciate the outcomes as well as to prevent scientific information to become inadvertently skewed to positive results.

## So, does RDN have a future role to play in the treatment of our patients?

RDN has been shown to be highly effective in several animal models of hypertension and cardiac arrhythmias. More than a decade ago, a number of clinical trials with RDN in hypertension suggested an enormous effect, only to be followed by the disappointing results of a carefully conducted, large, sham-controlled study (SYMPLICITY-HTN‑1, -2, and -3). Without going too much into the details of the discussion at the time (type of antihypertensive medication, medication non-compliance, effectiveness of RDN devices, office blood pressure vs 24-hour blood pressure), one of the major drawbacks of the available RND devices at that time was the inability to measure the sympathetic nerve damage around the renal artery immediately after energy delivery. At present, the assessment of effectiveness of ablation devices primarily relies on animal models, human cadaver studies and renal norepinephrine levels. Similar to physiological assessment of flow and flow reserve in the coronary arteries, renal flow reserve may become useful in this setting [2]. Although this issue still has not been solved, recent randomised sham-controlled trials (DENERHTN, SPYRAL HTN-ON MED, SPYRAL HTN-OFF MED, RADIANCE HTN SOLO) have sparked renewed interest because they demonstrated clinically significant reduction of ambulatory blood pressure in comparison with control groups [3]. More effective RDN may be related to second-generation radiofrequency (RF) ablation catheter systems (including Symplicity Spyral RF-RDN, Medtronic Vascular, Santa Rosa, CA, USA), ultrasound catheter systems (including ReCor Medical, Palo Alto, CA, USA), and combining ablation in the proximal renal artery and peripheral branches [4].

## What about heart failure?

There is ample evidence from preclinical rodent and swine studies that RDN may ameliorate chronic heart failure, yet clinical studies are scarce [5]. The REACH-Pilot study comprised 7 patients and showed improvement in six-minute walking test, but without control arm. It was published in 2013 and was not followed by a larger trial [6]. Several clinical studies with sham-controls are in preparation or enrolling (UNLOAD-HFpEF; ClinicalTrials.gov Identifier: NCT05030987 and RE-ADAPT-HF; ClinicalTrials.gov Identifier: NCT04947670) and results are to be expected in the upcoming years.

Feyz and colleagues are to be commended for putting these data together and although the primary endpoint was neutral, as a positive note, a significant difference was observed in left ventricular end-diastolic diameter in the RDN group, and 26% of patients in the treatment group were in NYHA class I versus none in the control group. While we learn how to effectively modulate the renal and systemic sympathetic nerve activity, renal denervation may soon, like a phoenix, rise from its ashes.
